# Identification and functional analysis of novel *FZD4* mutations in Han Chinese with familial exudative vitreoretinopathy

**DOI:** 10.1038/srep16120

**Published:** 2015-11-04

**Authors:** Ping Fei, Xiong Zhu, Zhilin Jiang, Shi Ma, Jing Li, Qi Zhang, Yu Zhou, Yu Xu, Zhengfu Tai, Lin Zhang, Lulin Huang, Zhenglin Yang, Peiquan Zhao, Xianjun Zhu

**Affiliations:** 1 Department of Ophthalmology, Xinhua Hospital Affiliated to Shanghai Jiaotong University School of Medicine, 1665 Kongjiang Road, Shanghai, 200092, China; 2 Sichuan Provincial Key Laboratory for Human Disease Gene Study, Department of Laboratory Medicine, Hospital of the University of Electronic Science and Technology of China and Sichuan Provincial People’s Hospital, Chengdu, Sichuan 610072, China; 3 Medicine Information Center, School of Medicine, University of Electronic Science and Technology of China, Chengdu, Sichuan, China; 4 Sichuan Translational Medicine Hospital, Chinese Academy of Sciences, Chengdu, Sichuan, China

## Abstract

Familial exudative vitreoretinopathy (FEVR) is a hereditary eye disease characterized by defects in the development of retinal vessels. However, known genetic mutations can only explain approximately 50% of FEVR patients. To assess the mutation frequency of *Frizzled 4* (*FZD4*) in Chinese patients, we analysed patients with FEVR from 61 families from China to identify mutations in *FZD4* and to study the effects of identified mutations on FZD4 function. All coding exons and adjacent intronic regions of *FZD4* were amplified by polymerase chain reaction and subjected to Sanger sequencing analysis. Three mutations in the *FZD4* gene were identified in these families. Of these, two were novel mutations: p.E134* and p.T503fs. Both mutations involve highly conserved residues and were not present in 800 normal individuals. Each of these two novel *FZD4* mutations was introduced into wild-type *FZD4* cDNA by site-directed mutagenesis. Wild-type and mutant *FZD4* DNAs were introduced into HEK293 cells to analyse the function of FZD4 in Norrin-dependent activation of the Norrin/β-catenin pathway using luciferase reporter assays. Both the p.E134* and p.T503fs mutants failed to induce luciferase reporter activity in response to Norrin. Our study identified two novel *FZD4* mutations in Chinese patients with FEVR.

Familial exudative vitreoretinopathy (FEVR, OMIM 133780) is a hereditary disorder with abnormal retinal vascular development^1^. This disease is characterized by the premature arrest of vascularization in the peripheral retina, which may result in retinal neovascularization and tractional retinal detachment^2^. However, the clinical phenotypes of FEVR vary widely, from very mild symptoms to complete blindness, even within the same family.

FEVR is inherited as an autosomal dominant trait in most cases, but it can also be inherited as an autosomal recessive or X-linked trait. In most cases, mutations in *FZD4* (OMIM 604579), *LRP5* (OMIM 653506), *TSPAN12* (OMIM 613138) and *ZNF408* cause the autosomal dominant form of FEVR^3^,^4^,^5^,^6^,^7^, while mutations in *LRP5* and *TSPAN12* may occasionally cause an autosomal recessive form of FEVR^8^,^9^,^10^. Mutations in *NDP* may result in X-linked forms of FEVR^11^,^12^. The encoded proteins of *FZD4*, *LRP5*, *TSPAN12 and NDP* genes are components of the Norrin/β-catenin signalling pathway. In addition, mutations in *KIF11*, a gene recently identified to cause microcephaly, lymphedema, and chorioretinal dysplasia (MLCRD), can also lead to FEVR condition^13^.

Previous studies suggested that known FEVR mutations explain approximately 40–60% of the autosomal dominant forms of FEVR cases in different populations^7^,^8^,^14^,^15^,^16^,^17^,^18^. In this study, we screened for mutations in the *FZD4* gene in 61 Chinese families with an autosomal dominant form of FEVR and found two novel mutations. We demonstrate that these two mutations in *FZD4* lead to the loss of *FZD4* activity.

## Materials and Methods

### Patients and clinic

Study approval was obtained from the Institutional Review Board of the Xinhua Hospital of Shanghai Jiaotong University School of Medicine and the Institutional Review Board of the Hospital of the University of Electronic Science and Technology of China and Sichuan Provincial People’s Hospital. All work was carried out in accordance with the approved study protocol. Informed consent was obtained from all participants in this study. For minor participants, written consent was obtained from the parents. In total, sixty-one Han Chinese families at risk for inheriting FEVR in an autosomal dominant form participated in the study. All participants underwent careful ophthalmological examinations. All participants were diagnosed by a clinical ophthalmologist, geneticist, and paediatrician based mainly on fundus photographic and angiographic changes. The angiographic changes in the patients were examined by intravenous injection of fluorescein dye. In the 800 normal matched controls, all individuals underwent an eye examination, and no signs of eye disease were observed.

### Mutation screening

Peripheral blood was collected from patients with FEVR and normal control subjects. Genomic DNA was isolated using a Qiagen genomic extraction kit following the manufacturer’s instructions. PCR primers were designed to include flanking intronic sequences of each exon of the *FZD4* gene (Supplementary Table 1). All coding regions were analysed via direct sequencing of PCR products. Amplified products were purified using a QIAquick Gel Extraction Kit (QIAGEN, Valencia, CA, USA) and sequenced with forward and reverse primers using a BigDye® Terminator v3.1 Cycle Sequencing Kit (ABI Applied Biosystems, Foster City, CA, USA) according to the manufacturer’s instructions. The sequences of the patients and the consensus sequences from the NCBI database were aligned using the DNAMAN program. The mutations were named following the recommendations of the Human Genomic Variation Society (HGVS).

### Bioinformatics analysis

Multiple protein sequence alignments of FZD4 proteins with their orthologues were generated using the ClustalW program provided by EMBL-EBI of the European Bioinformatics Institute (http://www.ebi.ac.uk/clustalw) to assess whether an amino acid substitution at the mutation position was evolutionarily conserved. Prediction of the possible effect of missense variants on the function of FZD4 protein was performed using SIFT and PROVEAN software.

### Construction of expression plasmids

*LRP5*, *FZD4* and *Norrin* expression vectors (generously provided by Dr. Jeremy Nathans of Johns Hopkins University, USA) have been previously described^19^. All mutations were introduced into the wild-type *FZD4* cDNA by site-directed mutagenesis using a QuikChange® Lightning Site-Directed Mutagenesis Kit (Agilent Technologies, Santa Clara, CA, USA). The recombinant plasmids containing FZD4-Flag fusion constructs were first verified by DNA sequencing and then prepared for transfection using a Qiagen plasmid Maxi preparation kit (QIAGEN, Valencia, CA, USA).

### Luciferase assays

The SuperTopFlash (STF) reporter, in which firefly luciferase is driven by 7 LEF/TCF consensus binding sites, was a kind gift from Dr. Jeremy Nathans. This reporter plasmid was stably transfected into HEK293 cells as previously reported to generate the STF cell line^19^. In 24-well plates, 160,000 STF cells/well were transfected with 800 ng DNA and 1.5 μL Lipofectamine^TM^ 2000 Transfection Reagent (Invitrogen, Carlsbad, CA, USA). The DNA mix contained 200 ng of Norrin, 200 ng of FZD4 (wild type or mutated), 200 ng of LRP5, and 100 ng of pSV-β-Galactosidase Control Vector. At 48 hours after transfection, cells were harvested and washed twice with PBS, and luciferase activities were measured with a Dual-Luciferase Assay Kit (Promega) according to the manufacturer’s instructions. Reporter activity was normalized to the coexpressed β-galactosidase activity in each well. Each test was performed in triplicate. This reporter assay was repeated three times, and a representative result is shown.

## Results

**Figure 1:**
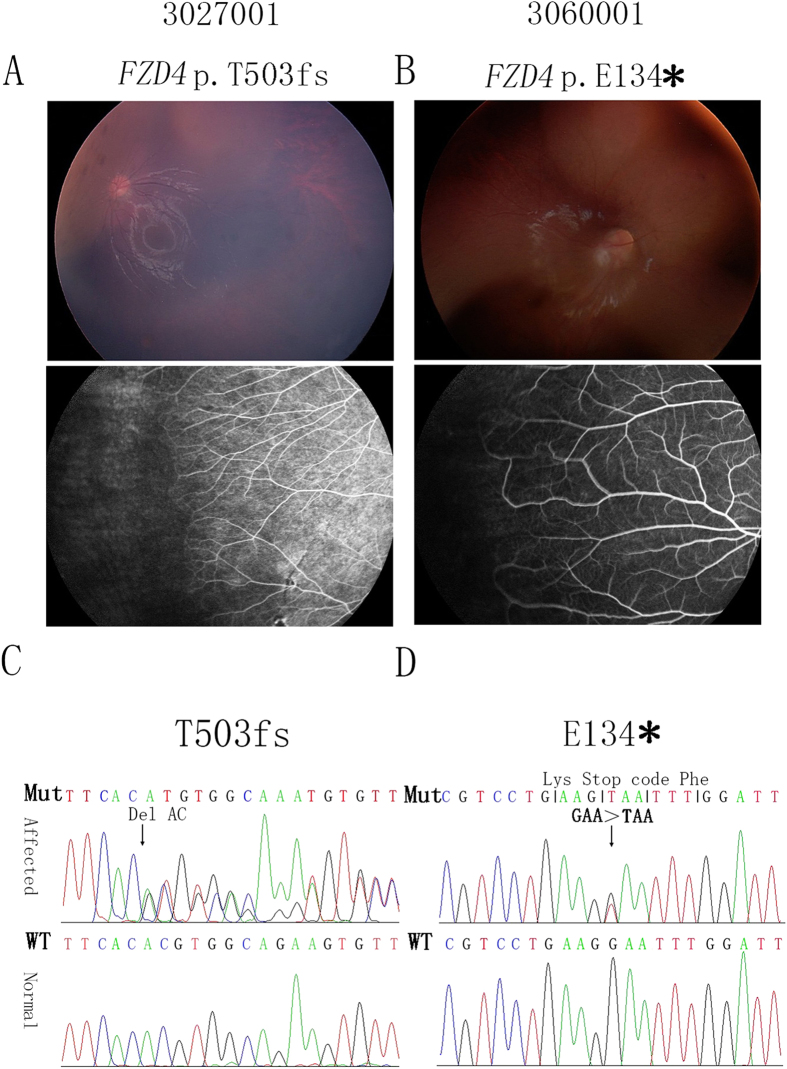
Ocular and angiographic changes and mutations in the FZD4 gene in families with FEVR. The individual ID and identified mutation are indicated at the top of each picture. Brush-like peripheral vessels were a typical sign of FEVR ((**A,B**) lower panel). Sequence chromatograms from patients and normal controls are shown in (**C,D**). Patient 3027001 carried a c.T1506delAC (p.T503fs) mutation (**C**), and patient 3060001 carried a c.G400T (p.E134*) mutation (**D**).

**Figure 2:**
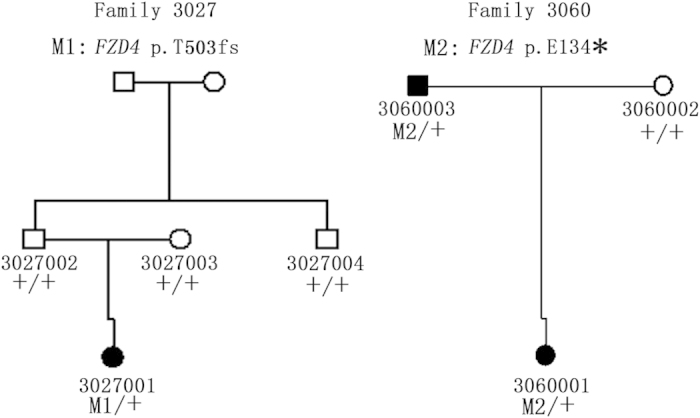
Pedigrees of families with autosomal dominant forms of FEVR who carried mutations identified in FZD4. (**A**) In pedigree 3027, subjects 3027002, 3027003 and 3027004 were negative for FEVR according to angiogram analysis. Consistent with the clinical results, none of the subjects carried the c.T1506delAC mutation (Supplemental Fig. 1). Therefore, this mutation was a *de novo* mutation in patient 3027001. (**B**) In pedigree 3060, subject 3060002 exhibited normal eyesight and showed no FEVR like symptoms, but angiogram analysis revealed defective peripheral retinal vessel development and brush-like peripheral vessels in both eyes. Sequencing analysis showed that he carried one copy of the c.G400T (p.E134*) mutation. The patient’s mother did not carry this mutation.

In this study, we screened for mutations in the *FZD4* gene in 61 Chinese families with an autosomal dominant form of FEVR by using PCR amplification and sequence analysis of all coding regions and flanking intronic regions. Among the 61 families with an autosomal dominant form of FEVR, we identified three mutations in the *FZD4* gene in three families, which accounted for 5% of all individuals (Supplementary table 2). Among these mutations, c.C205T(p.H69Y) was a known *FZD4* mutation^20^. The other two were novel mutations, c.T1506delAC (p.T503fs) in patient 3027001 and c.G400T (p.E134*) in patient 3060001 (Fig. 1). Both mutations co-segregated with the disease phenotype of the respective families (Fig. 2) and were absent in 800 normal controls. We then compared these two variants with the dbSNP135, 1000 Genomes project, HapMap project, YH database and a house-keeping database, which was generated by our lab with 2600 whole exome sequencing data. Both mutations were absent in these databases. We checked for the p.E134* and p.T503fs mutations in the human gene mutation database (h ttp://www.hgmd.org/) and found that the mutation is novel. We also checked for the mutation in the newly available ExAC database of 63,000 control exomes (http://exac.broadinstitute.org/), and no variants were reported in these loci of the *FZD4* gene.

**Figure 3:**
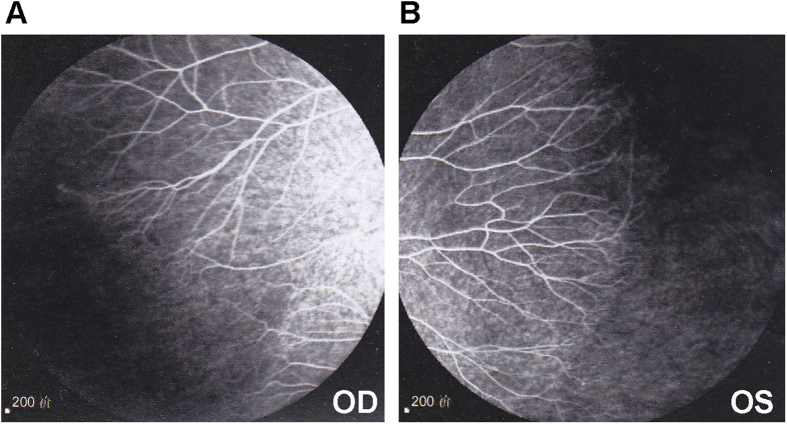
Angiographic changes in the asymptomatic mother of patient 3060001. The subject’s eyesight was normal, while FFA showed peripheral non-perfusion areas, increased ramification and brush-like peripheral vessels in both eyes.

Patient 3027001 was a two-year-old girl. Her right eye manifested total retinal detachment complicated by secondary glaucoma and cataracts. Her left eye showed retinal folds (Fig. 1A). The family history was negative. Sequencing analysis of additional family members showed that mutation c.T1506delAC (p.T503fs) was a *de novo* mutation (Supplemental Fig. 1). Patient 3060001 was a three-year-old girl. Her right eye was diagnosed with cataracts and a vitreous haemorrhage. After combined lensectomy and vitrectomy, a dragged disc was revealed. Her left eye showed peripheral avascular zones (Fig. 1D). Her father had normal eyesight, while FFA showed peripheral non-perfusion areas, increased ramification and brush-like peripheral vessels in both eyes (Fig. 3).

The p.E134* mutation, located in exon 2, changed the encoded residue from a glutamic acid, which is conserved among vertebrates, to a stop codon at codon 134, leading to a truncated protein missing two thirds of the c-terminal region. This mutation likely disrupts the function of FZD4. The p.T503fs mutation caused a frameshift change to the transcript at codon 503, which encodes a highly conserved threonine, leading to the production of a truncated protein.

**Figure 4:**
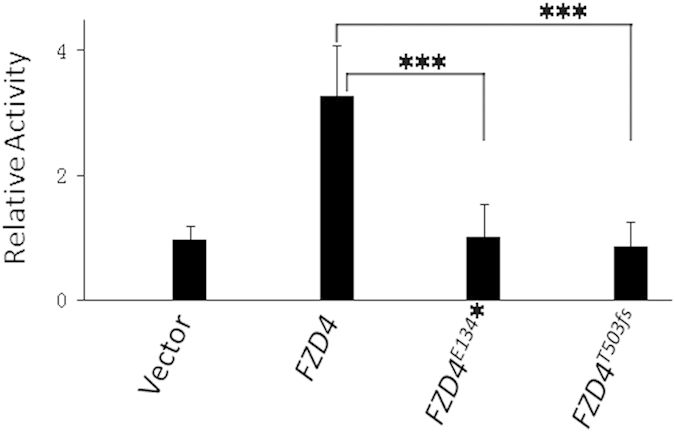
Mutant FZD4 proteins failed to activate the Wnt pathway. STF cells co-transfected with LRP5-pRK5 and pCMV6-FZD4 constructs were treated with Norrin and assayed for luciferase reporter activity. Neither of the two mutants of *FZD4* showed discernible activation of the luciferase reporter. Luciferase assays were performed in triplicate, and the results are shown as an average of four measurements. The difference between the mutant and wild type luciferase activity was statistically significant and marked by ***, as judged by a pairwise Student’s *t*-test (p < 0.05).

To evaluate the impact of these two novel mutations on FZD4 protein function, we introduced the corresponding mutations into *FZD4* cDNAs using a site-directed mutagenesis kit and analysed the function of mutant FZD4 proteins by using a Wnt-responsive firefly luciferase reporter system. Under physiological conditions, a complex of Norrin, FZD4, and LRP5 activates canonical Norrin/β-catenin signalling. As shown in Figure 4, both *FZD4* mutants failed to induce luciferase reporter activity in STF cells in response to Norrin (Fig. 4), confirming that the two mutations identified in our study abolish FZD4 function.

## Discussion

In our patients, the clinical phenotype of FEVR varied from asymptomatic to severe bilateral legal blindness. We have observed that FEVR in clinically asymptomatic patients can be detected using fluorescein angiography. The penetrance should be higher than originally thought based on only the clinical symptoms. Therefore, it is important to screen family members at the molecular level for FEVR mutations to reach a better diagnosis.

Previous studies indicated that mutations in known genes account for 50% of the autosomal dominant forms of FEVR cases in Caucasians, 40% in Japanese and 25% in Han C hinese^6^,^14^,^15^,^16^,^17^,^18^,^21^,^22^. In the current study, we identified two novel mutations in the *FZD4* gene that are responsible for FEVR in Han Chinese, and we demonstrated that these mutations are in conserved regions of the *FZD4* gene in vertebrates and lead to non-functional proteins.

Previous studies have demonstrated that *NDP, FZD4, LRP5* and *TSPAN12* responsible for FEVR are in the *NORRIN/*β-catenin signalling pathway and that the FEVR disease is caused by mutations of components in this pathway and *ZNF408*^19^,^23^,^24^,^25^. NDP is a ligand of FZD4, and FZD4, LRP5 and TSPAN12 together form a complex that activates the downstream Wnt pathway^25^,^26^. However, currently known mutations for FEVR can only explain 40–60% of all cases, indicating that there are additional gene(s) responsible for this disorder that have not yet been identified. Identifying other FEVR disease-causing genes and exploring their relationships with the *NORRIN/*B-catenin signalling pathway will provide insight and further understanding into the pathogenesis of FEVR^27^.

## Additional Information

**How to cite this article**: Fei, P. *et al.* Identification and functional analysis of novel *FZD4* mutations in Han Chinese with familial exudative vitreoretinopathy. *Sci. Rep.* **5**, 16120; doi: 10.1038/srep16120 (2015).

## Supplementary Material

Supplementary Dataset
