# Supplementary material for: Identification and functional analysis of novel FZD4 mutations in Han Chinese with familial exudative vitreoretinopathy
Source: Sci Rep. 2015 Nov 4;5:16120. doi: 10.1038/srep16120 (PMC4632081; doi:10.1038/srep16120)
Supplement: Supplementary Dataset [file srep16120-s1.pdf]

Identification and functional analysis of novel *FZD4* mutations in Han Chinese with familial exudative vitreoretinopathy

Ping Fei[1]*, Xiong Zhu[2,3,4*], Zhilin Jiang[2,3,4]*, Shi Ma[2,3], Jing Li[1], Qi Zhang[1], Yu Zhou [2,3,4], Yu Xu[1], Zhengfu Tai[2,3,4], Luling Huang[2,3,4], Zhenglin Yang[1,2,3,4], Peiquan Zhao[1], Xianjun Zhu[2,3,4]

[*]These authors contributed equally to this study.

Supplementary information file

Supplementary information file includes a table (**Table S1**) and a figure (Figure S1).

Table S1. Sequencing primers for *FZD4* gene.

Table S2. Mutations in *FZD4* identified in Chinese Han Patients with FEVR in this study.

Figure S1. *FZD4* genotypes of additional members of family 3027. A, in pedigree 3027, subjects 3027002, 3027003 and 3027004 are negative for FEVR by angiogram analysis. In consistent with the clinical results, all of them didn't carry the c.T1506delAC mutation. Therefore, this mutation is a de nova mutation in patient 3027001.

Table S1. Sequencing primers for *FZD4* gene

Primer name	Sequence
FZD4-EXON1-F	GGGACGTCTAAAATCCCACA
FZD4-EXON1-R	CTCCTTCGGGCTAGGATGAT
FZD4-EXON2-1-F	ACTCAGCTTTGTGGGAGCAT
FZD4-EXON2-1-R	AGGCTCCTTTTCACCCAGAT
FZD4-EXON2-2-F	TGCCCTTACCTCACAAAACC
FZD4-EXON2-2-R	AGGTTCTGCTGCCTCTTCAA
FZD4-EXON2-3-F	CAGCCTGTGTTTCATCTCCA
FZD4-EXON2-3-R	AATCACACACGTTGCAGGAA
FZD4-EXON2-4-F	CTGGCTTGTGCTATGTTGGA
FZD4-EXON2-4-R	CGGGGGTCACTTAATTGTTG

Table S2. Mutations in *FZD4* identified in Chinese Han Patients with FEVR in this study.

Patient Number	Gene/exon	DNA Change	Protein Change	Allele Status	Note
3501001	*FZD4/2*	c.C205T	p.H69Y	Hetero	Known[1]
3060001	*FZD4/2*	c.G400T	p.E134X	Hetero	Novel
3027001	*FZD4/2*	c.1602delA[illegible]	p.T515fs	Hetero	Novel

[1] Kondo H, Kusaka S, Yoshinaga A, Uchio E, Tawara A, Tahira T. Genetic variants of FZD4 and LRP5 genes in patients with advanced retinopathy of prematurity. Mol Vis. 2013;19:476-85.

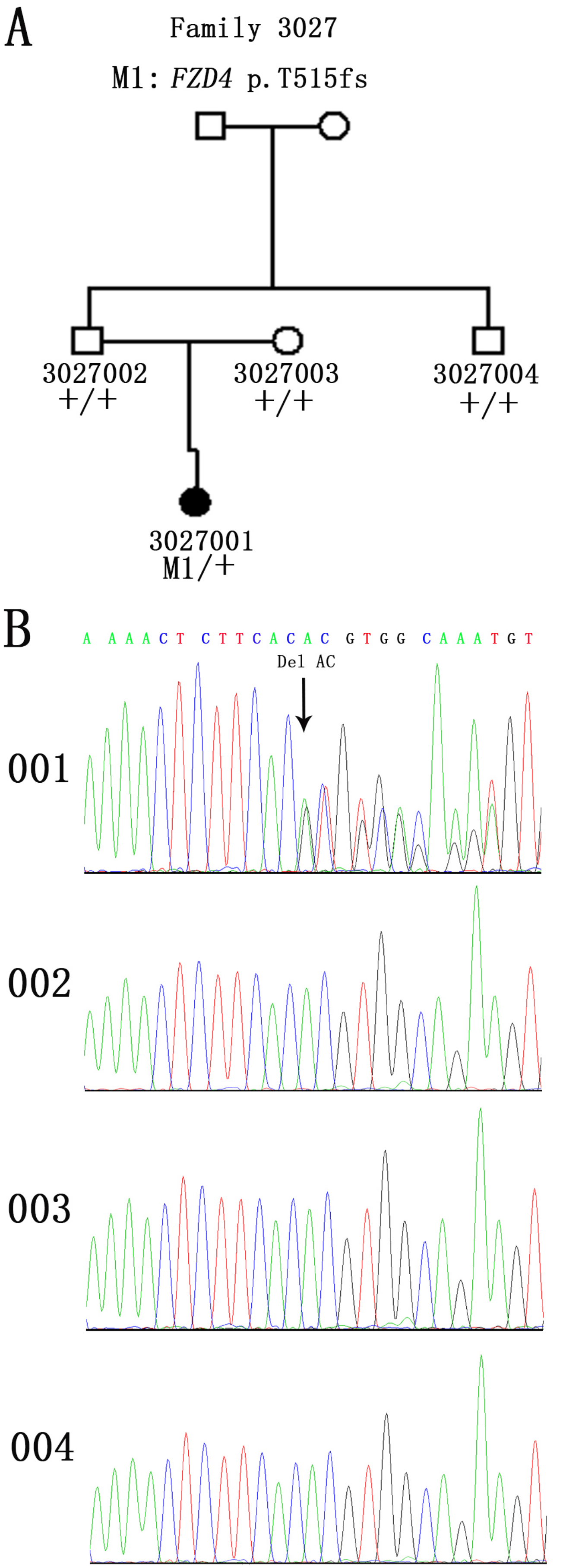
A
Family 3027
M1: FZD4 p.T515fs
3027002
+/+
3027003
+/+
3027004
+/+
3027001
M1/+
B
A AAACT CTTCACAC GTGG CAAATGT
Del AC
001
002
003
004
